# Continuing professional education of Iranian healthcare professionals in shared decision-making: lessons learned

**DOI:** 10.1186/s12913-021-06233-6

**Published:** 2021-03-12

**Authors:** Samira Abbasgholizadeh Rahimi, Charo Rodriguez, Jordie Croteau, Alireza Sadeghpour, Amir-Mohammad Navali, France Légaré

**Affiliations:** 1 Department of Family Medicine, Faculty of Medicine and Health Sciences, McGill University, Montréal, Canada; 2 Lady Davis Institute for Medical Research, Jewish General Hospital, Montréal, Canada; 3 Institute of Health Sciences Education (IHSE), Faculty of Medicine and Health Sciences, McGill University, Montréal, Canada; 4 L’institut national d’excellence en santé et en services sociaux (INESSS), Quebec City, Canada; 5 Tabriz University of Medical Sciences, Tabriz, Iran; 6 Orthopedic Surgery Department, Shohada University Hospital, Tabriz, Iran; 7 Department of Family Medicine and Emergency Medicine, Faculty of Medicine, Université Laval, Quebec City, Canada; 8 VITAM – Centre de recherche en santé durable, Centre intégré universitaire de santé et services sociaux de la Capitale-Nationale, Quebec City, Canada

**Keywords:** Shared decision making, Patient engagement, Continuous professional development, Medical education, Iran, Implementation

## Abstract

**Background:**

In this study, we sought to assess healthcare professionals’ acceptance of and satisfaction with a shared decision making (SDM) educational workshop, its impact on their intention to use SDM, and their perceived facilitators and barriers to the implementation of SDM in clinical settings in Iran.

**Methods:**

We conducted an observational quantitative study that involved measurements before, during, and immediately after the educational intervention at stake. We invited healthcare professionals affiliated with Tabriz University of Medical Sciences, East Azerbaijan, Iran, to attend a half-day workshop on SDM in December 2016. Decisions about prenatal screening and knee replacement surgery was used as clinical vignettes. We provided a patient decision aid on prenatal screening that complied with the International Patient Decision Aids Standards and used illustrate videos. Participants completed a sociodemographic questionnaire and a questionnaire to assess their familiarity with SDM, a questionnaire based on theoretical domains framework to assess their intention to implement SDM, a questionnaire about their perceived facilitators and barriers of implementing SDM in their clinical practice, continuous professional development reaction questionnaire, and workshop evaluation. Quantitative data was analyzed descriptively and with multiple linear regression.

**Results:**

Among the 60 healthcare professionals invited, 41 participated (68%). Twenty-three were female (57%), 18 were specialized in family and emergency medicine, or community and preventive medicine (43%), nine were surgeons (22%), and 14 (35%) were other types of specialists. Participants’ mean age was 37.51 ± 8.64 years with 8.09 ± 7.8 years of clinical experience. Prior to the workshop, their familiarity with SDM was 3.10 ± 2.82 out of 9. After the workshop, their belief that practicing SDM would be beneficial and useful (beliefs about consequences) (beta = 0.67, 95% CI 0.27, 1.06) and beliefs about capability of using SDM (beta = 0.32, 95% CI -0.08, 0.72) had the strongest influence on their intention of practicing SDM. Participants perceived the main facilitator and barrier to perform SDM were training and high patient load, respectively.

**Conclusions:**

Participants thought the workshop was a good way to learn SDM and that they would be able to use what they had learned in their clinical practice. Future studies need to study the level of intention of participants in longer term and evaluate the impact of cultural differences on practicing SDM and its implementation in both western and non-western countries.

**Supplementary Information:**

The online version contains supplementary material available at 10.1186/s12913-021-06233-6.

## Background

Shared decision making (SDM) is a collaborative process in which patients and healthcare professionals make healthcare decisions based on the best available evidence and on patients’ priorities [1]. It is now commonly accepted that patients should be adequately informed about the potential risks and benefits of different treatment options, including medications such as their respective health impacts in the long-term. To make health-related decisions, patients first need to identify what is most important to them, then, they need to be supported in their decision-making [2]. Patients involved in decisions about their health, report better experiences of care and obtain better health outcomes [3]. However, achieving the full potential of SDM, including greater patient involvement, requires that healthcare professionals have the attitudes and skills necessary to predispose them to SDM [4].

In western countries, governments endorse SDM through healthcare policy and regulations [5, 6], and a few countries have begun to provide SDM education to healthcare professionals and trainees. Healthcare professionals’ implementation of SDM in their practice has been slow [4]. With respect to non-western countries, a systematic review showed that patient participation in decision making can be feasible and effective, provided healthcare professionals receive training [7]. However, very little training is provided. In Iran, for example, despite recent developments favoring informed decision making in the healthcare system and despite increasing attention to patient-centered care, SDM knowledge and training is not widespread [8].

### Shared decision-making in non-western countries, such as Iranian

Culture, an abstract and complex concept, constantly influences different dimensions of human behavior, [9] including decision-making during medical encounters. The Iranian population is made up of many ethnic groups whose respective cultural norms are reflected in the country’s overarching culture. Thus, for innovations to be accepted, geographical, political, historical, cultural and social dimensions must be considered [10]. Moreover, assessing and addressing cultural differences are important in health communications, including medical decision-making communications [11] and in implementation of SDM. For instance, in eastern countries, such as Iran, the culture is one of collectivism compared to the individualism of the west [11]. Thus, patients may value having loved ones (e.g., family and friends) accompany them when a cancer diagnosis is given [12]. Moreover, as in many Asian countries, patients are “protected” from bad news in Iran, such as negative consequences of their health conditions, during the medical encounter [13, 14]. As a result, healthcare professionals either counsel patients’ family members regarding medical decisions or they make the decisions for the patients. Overall, given cultural differences, what works when implementing SDM in western countries is not what necessarily will work in non-western countries such Iran.

Successful implementation of SDM requires taking into account individual psychological constructs such as perception of health and illness, attitude, concepts of power, and social networks, which may vary as a function of culture [11]. In Iran, patients are concerned with the social consequences of a health problem, such as consequences for their family (*Perception of health and illness*). Attitudes of patients are influenced by the context and integrate others’ views and norms, especially those of family members, since family ties are very important culturally (*Attitude*). Elders and those in authority, such as healthcare professionals, are highly respected and challenging their authority is considered disrespectful (*Concept of power*). Finally, patients are more likely to listen to the advice of their family or intimate peers than that of others (*Social networks)*. To implement and popularize the SDM model in non -western countries such as Iran, it may be essential to adapt it according to the above-mentioned cultural characteristics.

### Shared decision-making education in Iran

Most SDM education, implementation, and research have been restricted to a few high-income western countries [15]. Iran is a middle-income eastern country where SDM is poorly known, despite recent policy developments in support of informed decision making and increasing attention to patient-centered care [8]. Indeed, most Iranian healthcare professionals are unfamiliar with the principles of SDM. There are no published studies about training activities for SDM among Iranian primary healthcare professionals or other health professionals. Thus, few healthcare professionals have the skills to implement it in their clinical settings [8].

Workshops are among the most commonly used methods to provide training to busy healthcare professionals to improve their knowledge and skills [16]. However, workshop method is not necessarily what is traditional in Iran. We decided to break away from the traditional Iranian continuing professional medical education model, and design, implement and assess an SDM workshop. The questions that we evaluated during this educational innovation were: [1] To what extent do participants consider the SDM continuing professional development workshop acceptable and satisfactory? [2] To what extent does healthcare professionals’ participation in this workshop influence their intention to adopt SDM in their clinical practice? [3] According to participants, what are the facilitating factors and barriers that could influence the adoption of SDM in the Iranian healthcare system?

## Methods

### Study design and context

We conducted an observational study design with quantitative methods. The workshop was held at Tabriz University of Medical Sciences, East Azerbaijan, Tabriz, Iran in December 2016. Using email and text messages, we invited a purposeful sample of healthcare professionals, including trainees (*n* = 60) affiliated with the Tabriz University of Medical Sciences. No limitations were defined for age, ethnicity, gender, or years of clinical experience. The workshop was held in a conference room equipped with audio-visual technology. Ethical approval for the research component of the intervention was granted by Tabriz University of Medical Sciences, and prior to the workshop, all participants signed an informed consent form. This paper is written according to the reporting guideline for group-based behavior-change interventions [17] which was designed for behavior interventions, like ours, that are delivered by at least one facilitator to at least three participants (Additional file 1).

### The workshop: continuing professional educational intervention on SDM

The SDM workshop was a half day, designed to be interactive and adapted to Iranian cultural context. We validated it by both Iranians and Canadians who were experts in SDM, continuing professional development, knowledge translation, and implementation science.

### Workshop implementation

**Fig. 1:**
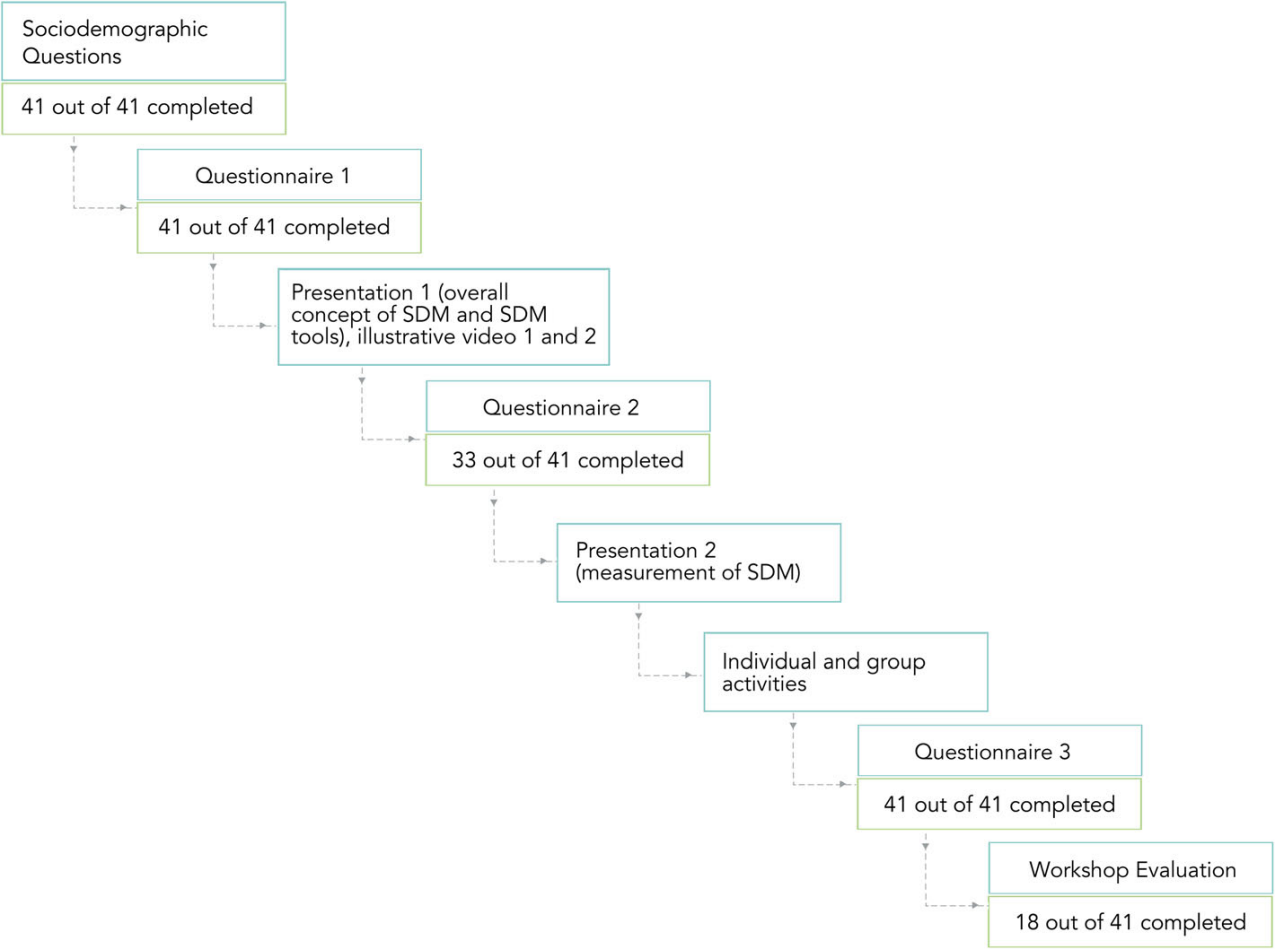
Detailed steps of workshop and numbers of completed questionnaires

The interactive workshop was held at the Tabriz University of Medical Sciences, East Azerbaijan, Tabriz, Iran (December 22, 2016). It was facilitated by the first author (SAR), who has expertise in SDM, and coordinated by two healthcare professionals from Tabriz University of Medical Sciences (AS, AMN). Neither the coordinators nor the facilitator had any previous relationships with the participants. The duration of the workshop designed to be 4 h, including a 15-min break. The content of the workshop had three foci: (1) overall concept of SDM, (2) SDM tools, and (3) measurement of SDM. It was delivered using an interactive combination of lectures individual and group activities (Fig. 1). For the lectures, instructional materials were PowerPoint slides and illustrative videos. For the activities, handouts were provided, namely a patient decision aid on prenatal screening for Down Syndrome [18], the International Patient Decision Aids Standards (IPDAS) checklist (for use in individual and group activities) [19].

The workshop was structured as follows. First, participants completed the sociodemographic questions and a questionnaire to evaluate their knowledge about SDM and intention to use it in clinical practice. Second, the overall concept of SDM (*focus 1*) was presented. Third, participants watched two videos that illustrated the behaviour (performing SDM in two clinical contexts). The first showed an orthopedic surgical patient who was using an SDM process to make a decision about knee replacement surgery [20], and explaining his experience. The second video showed two simulated consecutive prenatal follow-up consultations during which a pregnant woman, her partner, and a healthcare professional used a PtDA about Down Syndrome prenatal screening [21, 22]. Other studies which, similarly, applied vignettes for explaining SDM, showed that healthcare professionals’ knowledge and intention to engage in SDM has increased [23]. Fourth, participants completed a questionnaire evaluating the 12 domains of TDF. Fifth, SDM measurement was presented. Sixth, participants were asked to individually evaluate a patient decision aid for prenatal screening using IPDAS checklist.

Seventh, participants were randomly divided into groups of three to six people. Participants were asked to either remember or imagine a situation in which they could practice SDM in their clinical setting and discuss it in their groups with guidance from the questions on the group activity handout. These questions were: When and where could you use SDM in your clinical setting? What are the potential advantages and disadvantages of practicing SDM in this situation? How could SDM practices be facilitated in your clinical setting? What are the barriers?

### Data collection

For data collection, at the beginning of the workshop, we used a sociodemographic questionnaire and a self-report questionnaire to assess their familiarity with SDM. Then we used a questionnaire based on the Theoretical Domains Framework (questionnaire 1). This framework is composed of 12 theoretical domains relevant to behavior change [24]: 1) knowledge, 2) skills, 3) social/professional role and identity (self-standards), 4) beliefs about capabilities (self-efficacy), 5) beliefs about consequences (Anticipated outcomes/attitude), 6) motivation and goals (intention), 7) memory and attention, 8) environmental context and resources (environmental constraints), 9) social influences (norms), 10) emotion 11) behavioral regulation, and 12) nature of the behaviors. We defined behavior as follows: performing SDM (action) among Iranian health professionals (target) in any clinical setting (context).

During the workshop activities, participants also responded a questionnaire about their perceived facilitators and barriers of implementing SDM in their clinical practice (questionnaire 2). The workshop concluded with participants completing two questionnaires i.e., continuous professional development (CPD) reaction questionnaire (questionnaire 3), and workshop evaluation questionnaire. The first, adapted from previous work in the field, assessed the impact of the workshop, i.e. to evaluate whether healthcare professionals were likely to implement what they learned [25]. This questionnaire is based on constructs (i.e. intention, social influences, beliefs about capabilities, moral norm, and beliefs about consequences) [25] and scored on a 7-point Likert scale, except for one question which is scored on a 5-point percentage scale. The second, evaluated the content, design, instructor, and results of the workshop via nine questions scored on a 5-point Likert scale. This questionnaire included space for participants to provide suggestions to help us further improve the workshop. Figure 1 shows the detailed steps of the workshop and questionnaires.

### Data analysis

All analyses were performed using R version 3.4.3. We first calculated descriptive statistics on the participants’ responses. We used the mean with standard deviation for continuous and ordinal variables (age, experience, theoretical domains, and workshop evaluation questions) and frequency with percentage for categorical variables (gender, type of healthcare professional, prior knowledge about SDM, facilitators and barriers). Then we performed multiple linear regression of the healthcare professionals’ intention on the psychological constructs together in the same model. This allowed us to estimate the influence of each construct on intention to use SDM while adjusting for the other constructs. The data related to the facilitators and barriers were coded by two researchers, and their frequency and percentage were calculated.

## Results

### Participant characteristics

Of the 60 healthcare professionals who were invited to the workshop, 41 participated (68%). Their characteristics are shown in Table 1. Figure 1 shows the detailed steps of the workshop and numbers of completed questionnaires. All of the participants completed sociodemographic questionnaire, and questionnaire to assess their familiarity with SDM. Before the workshop, 70% of the participants (*n* = 29) had prior knowledge about SDM, reporting familiarity with SDM of 3.1 ± 2.82, on average, out of 9. However, they had very little experience using it in their practice (Fig. 1).

**Table 1:** Participant characteristics and prior knowledge of SDM, *N* = 41

Characteristic	Value
***Age (years), mean (SD)***	37.51 (8.64)
***Experience (years), mean (SD)***	8.09 (7.80)
***Sex, n (%)***	
Male	18 (43)
Female	23 (57)
***Type of healthcare professional, n (%)***	
Family, emergency, community/preventive medicine or internal medicine	18 (43)
Surgery or anesthesiology	9 (22)
Others (healthcare professionals/trainees in gynecology, otolaryngology, pathology, radiology, dermatology, neurology, and psychiatry)	14 (35)
***Prior knowledge of shared decision making, n (%)***	
Yes	29 (70)
No	12 (30)

All of the participants completed questionnaire 1. The level of intention for practicing SDM among healthcare professionals was high (5.51 ± 1.35 out of 7). Participants believed the consequences of practicing SDM would be very beneficial and useful (beliefs about consequences) (5.71 ± 1.14 out of 7); they also believed practicing SDM would be highly acceptable and in accordance with Iranian moral values (moral norm) (5.56 ± 1.28 out of 7) (Table 2).

**Table 2:** Theoretical Domains Framework, psychological factors

Construct	Mean (out of 7)	SD
Intention	**5.51**	1.35
Social influences	4.20	1.21
Beliefs about capabilities	5.01	1.05
Moral norm	**5.56**	1.28
Beliefs about consequences	**5.71**	1.14

Beliefs about consequences (beta = 0.67, 95% CI 0.27, 1.06) had the strongest influence on participants’ intention to practice SDM (Table 3). Their beliefs about consequences were strongly correlated with their intention, even after adjustment for the other constructs. Beliefs about capabilities (beta = 0.32, 95% CI -0.08,0.72) had the second strongest effect, but was not significant at 5% (Table 3).

**Table 3:** Significant factors in participants’ intentions to practice SDM in their clinical settings

Construct (correlated with intention)	Beta	***P***
Social influences	−0.03 (− 0.27, 0.21)	0.810
Beliefs about capabilities	0.32 (−0.08, 0.72)	0.120
Moral norm	0.10 (−0.20, 0.40)	0.520
Beliefs about consequences	**0.67 (0.27, 1.06)**	0.002

Out of 41 participants 33 completed questionnaire 2. Thirteen answered question related to the facilitators and 33 answered question related to the barriers. The most frequently mentioned facilitators were “training” (23%) and “managerial support” (15%), “patients’ or healthcare professionals’ motivation” (15%) and “availability of environmental resources” (15%). The most frequently mentioned barriers were “High patient load” (33%) and “time constraints” (31%) (Table 4).

**Table 4:** Perceived facilitators and barriers to practice SDM

Facilitators	Frequency ***n*** (%)
Training (healthcare professionals and patients)	3 (23)
Managerial support	2 (15)
Patients’/healthcare professionals’ motivation	2 (15)
Availability of environmental resources	2 (15)
Development of web-based training	1 (8)
Availability of decision aids	1 (8)
Favorable moral norms	1 (8)
Good socioeconomic status (hospitals/patients)	1 (8)
**Barriers**	***n*** **(%)**
High patient load	11 (33)
Time constraints	10 (31)
Lack of knowledge and education among patients	5 (15)
Lack of environmental resources	3 (9)
Cultural barriers and ethical issues	3 (9)
Resistance to change among healthcare professionals	1 (3)

All of the participants completed questionnaire 3, and 18 completed workshop evaluation questionnaire. The results of questionnaire 3 and workshop evaluation are shown in Table 5.

**Table 5:** CPD reaction questionnaire and workshop evaluation

CPD Reaction Questionnaire	Mean (SD)
I intend to perform SDM (strongly agree = 1— strongly disagree =7)	5.61 (1.45)
To the best of my knowledge, the percentage of my colleagues whouse SDM is: (0–20%, 20–40%, 41–60%, 61–80%,81–100%)	Frequency of 0–20% = 12
	Frequency of 20–40% = 7
	Frequency of 41–60% = 9
	Frequency of 61–80% = 12
	Frequency of 81–100% = 1
I am confident that I could perform SDM if I wanted to.(strongly agree = 1— strongly disagree =7)	5.17 (1.56)
Performing SDM is the ethical thing to do.(strongly agree = 1— strongly disagree =7)	5.66 (1.61)
For me, performing SDM would be: (extremelydifficult = 1—extremely easy = 7)	4.63 (1.28)
Now think about a co-worker whom you respect as a professional.In your opinion, does he/she perform SDM? (never = 1— always =7)	4.46 (1.38)
I plan to perform SDM. (strongly agree = 1— strongly disagree =7)	5.41 (1.48)
Overall, I think that for me performing SDM would be: (useless = 1— useful =7)	5.58 (1.41)
Most people who are important to me in my profession perform SDM.(strongly agree = 1— strongly disagree =7)	4.51 (1.61)
It is acceptable to perform SDM. (strongly agree = 1— strongly disagree =7)	5.46 (1.42)
I have the ability to perform SDM. (strongly agree = 1— strongly disagree =7)	5.22 (1.39)
Overall, I think that for me using SDM would be: (harmful = 1—beneficial = 7)	5.83 (1.6)
**Workshop evaluation**	**Mean (SD) out of 5**
Was well-informed about the objectives of this workshop	4.17 (0.72)
Thought the workshop met their expectations	4.00 (0.94)
Thought the content were relevant to their job	4.20 (0.90)
Thought the workshop objectives were clear to them	4.65 (0.61)
Thought the workshop activities were stimulating	4.18 (0.81)
Thought the instructor was well prepared	4.35 (0.70)
Thought the instructor was helpful	4.41 (0.71)
Thought they would be able to use what they learned in the workshop	4.06 (0.82)
Thought the workshop was a good way for them to learn this content	4.18 (0.64)

## Discussion

In this paper, we describe the delivery and evaluation of an SDM workshop at Tabriz University of Medical Sciences, East Azerbaijan, Tabriz, Iran. We evaluated the acceptability of and satisfaction with the workshop **(aim 1)** as well as its impact on participants’ intention to practice SDM in their clinical setting **(aim 2)**. Moreover, we explored participants’ perceived facilitators and barriers to practice SDM in their clinical setting **(aim 3)**. The results of this study suggest the following:

***First (aim 1),*** overall, the SDM workshop was highly acceptable to the participating Iranian healthcare professionals and trainees. They found that the workshop met their expectations, was relevant to their job, and stimulating. Further, they found the workshop as a good way to learn SDM and thought they would be able to use what they had learned in their clinical practice.

***Second (aim 2),*** the intention to use SDM was high among the participants. A previous study with 299 Iranian patients, showed a high level of intention to be informed about and involved in health decisions [14]. Together, these results suggest that both Iranian healthcare professionals and patients may be inclined to engage in SDM, and their lack of intention may not be the reason for the lack of SDM implementation in Iran. However, additional research with larger numbers of participants will be needed to reach to this conclusion.

Beliefs about consequences of practicing SDM—knowing that it could be beneficial and useful—was highly correlated with participants intention to practice SDM in their clinical setting. Therefore, reinforcing Iranian healthcare professionals’ beliefs about positive consequences of SDM could have a positive effect on their attitudes and consequently the implementation of SDM in their clinical practice. Strategies such as increasing healthcare professionals’ knowledge about SDM and showing them its benefits and usefulness could facilitate its implementation in Iran. This could be done through seminars, online training, and other types of educational activities. Further, using examples from inside the country could not only reassure healthcare professionals’ about the feasibility of performing SDM in Iran, but also could be motivational.

***Third (aim 3),*** participants mentioned training (of both healthcare professionals and patients) and their motivation as facilitators to performing SDM, and their limited knowledge about SDM and lack of patient SDM education as barriers of practicing SDM in their clinical practice. These results are aligned with those of a recent systematic review on patient’s involvement in decision-making in non-western countries that highlighted the need for healthcare professional and patient training in SDM [7]. Patients lack of familiarity with their rights and their low health literacy were acknowledged as barriers in other Iranian studies, as well [23, 26]. However, further studies are required regarding which training format could work best for healthcare professionals and patients in non-western countries such as Iran, and what are the impact of these trainings on SDM implementation in long term.

Participants also mentioned managerial support, availability of environmental resources, good socioeconomic status, and availability of SDM tools (such as decision aids) as other facilitators of practicing SDM in their clinical practice. Additional barriers mentioned were high patient load, time constraints, and lack of environmental resources. This is similar to what Légaré and colleagues found as the barriers of SDM in home healthcare services [27]. The government of Iran could facilitate implementation of SDM by providing the needed managerial support and resources to clinical settings and by encouraging research in this area. Policy makers can play an important role in overcoming these perceived barriers and in stimulating these perceived facilitators. SDM research and practice should also be emphasized at the policy level, including governmental guidelines and regulations [8].

Participants’ cultural boundaries and resistance to change were also highlighted in our study as barriers to implementing SDM in clinical practice. Attitudes of patients considering their collective culture is often influenced by family and intimate peers’ views and norms, and they are likely to approve of their messages than from anyone else. Therefore, since family ties are considered very important culturally, person-centric decision-making models in this culture might not be suitable. One strategy to put SDM in practice in such cultures could be to communicate and share the decision not only with patient but also with their family. Decision aids may need to be designed to support family involvement.

Other cultural barriers may be rooted in beliefs about authority and power in patient-physician relationships. For example, challenging elders and those in authority, such as healthcare professionals, is seen as disrespectful in Iran. Thus, some patients, especially older or more traditional ones, may refrain from asking questions. Healthcare professionals in Iran therefore need to be more intentional and encouraging about inviting patients to ask questions about their treatment or screening options, knowing that this may go against long-established social norms. This could also empower them to be more engaged in decision-making processes about their health in general. Given that we know SDM could lead to improved affective-cognitive outcomes [28], more investigation into cultural differences to adapt the SDM model to non-western countries, such as Iran, is required.

### Limitations of the study

First, a self-selection bias may be present in this study in that those who agreed to participate to the workshop may have been more inclined to patient-centered care and SDM than those who did not respond to our invitation. Second, the invitation to participate in the workshop was sent to organizations and personal email lists. It is therefore possible that our invitation did not reach all eligible participants. Third, the study targeted healthcare professionals in one province of Iran, thus we cannot infer that our results are applicable to other provinces and territories; however, our workshop results and lessons learned can inspire future efforts. Lastly, our study does not explicitly measure the long-term impact of the workshop on skills and/or attitudes variables. We encourage future work to evaluate the long-term impact of such educational activities on participants intention to use SDM in non-western contexts.

## Conclusion

Our half-day workshop among healthcare professionals in an Iranian context was found to be acceptable and satisfying. Participants found that the workshop was a good way to learn SDM, and that they would be able to use what they had learned in their clinical practice. Participants’ intention to practice SDM was high following the workshop, and the belief that practicing SDM would be beneficial and useful (beliefs about consequences) had the strongest influence on their intention to practice SDM. The broader implication of our study is to inform future SDM educational activities in non-western and western countries, and to encourage further studies to evaluate the impact of cultural differences on practicing SDM and its implementation in both western and non-western countries.

## Supplementary Information

**Additional file 1: ** The reporting guideline for group-based behavior-change interventions: continuing professional education of Iranian healthcare professionals in shared decision making.

## Data Availability

The materials in the manuscript are freely available to researchers who wish to use them for non-commercial purposes, provided they preserve confidentiality and anonymity and cite the original reference in any publications.

## Abbreviations

SDM: Shared Decision Making
PtDA: Patient Decision Aid
IPDAS: International Patient Decision Aids Standards
TDF: Theoretical Domains Framework
