# Supplementary material for: Continuing professional education of Iranian healthcare professionals in shared decision-making: lessons learned
Source: BMC Health Serv Res. 2021 Mar 12;21:225. doi: 10.1186/s12913-021-06233-6 (PMC7953598; doi:10.1186/s12913-021-06233-6)
Supplement: Supplementary file 1 — Additional file 1: The reporting guideline for group-based behavior-change interventions: continuing professional education of Iranian healthcare professionals in shared decision making. [file 12913_2021_6233_MOESM1_ESM.docx]

| Essential reporting elements | Page |
| --- | --- |
| 1. Intervention source or development methods  (Describes the source (origin) and/or methods used for developing the intervention) | 4-6 |
| 2. General setting  (Reports the type of setting where the group sessions were delivered) | 6-7 |
| 3. Venue characteristics  (Describes the set up or configuration of the room (or other venue) where the group meetings took place.) | 6-7 |
| 4. Total number of group sessions  (The total number of group sessions in the program is reported or it is possible for this to be calculated) | NA |
| 5. Length of group sessions  (Reports the length of group sessions (average and/or range)) | 6 |
| 6. Frequency of group sessions  (Reports the frequency of group sessions, i.e., how often they were delivered) | NA |
| 7. Duration of intervention  (Reports the duration of the intervention, i.e., over what period of time the group sessions were delivered) | 6 |
| 8. Change mechanisms or theories  (Describes how the intervention was intended to work by identifying change mechanisms or underpinning theories of behavior change) | 7 |
| 9. Change techniques  (Describes the techniques used in group sessions to prompt change. These may be derived from the mechanisms or theories of change, and may use established taxonomies of behavior change) | 7 |
| 10. Session content  (Describes the content of the sessions in terms of themes or topics covered, i.e., what the sessions were about) | 6-7 |
| 11. Sequencing of sessions  (Indicates whether there is a logical (sequential) progression of session content or, alternatively, that the content of all sessions is the same, i.e., a repetitive, or “rolling”, program with no particular start or end point) | NA |
| 12. Participants’ materials  (Reports what materials or tools the participants used during and outside the group sessions.) | 6-7 |
| 13. Activities  (Describes what the participants and the facilitators did during group sessions, i.e., what happened during the sessions) | 6-7 |
| 14. Fidelity of session delivery  (Reports methods used to check the fidelity of intervention delivery, i.e., methods used to check if the sessions were delivered as designed.) | NA |
| 15. Group composition  (Provides information on the composition of the groups in the intervention, i.e., who were the participants in the groups or whether there were any differences in the participants’ characteristics between groups.) | 6-7 |
| 16. Methods for group allocation  (Describes methods used to allocate the participants to different groups) | 6-7 |
| 17. Continuity of participants’ group membership  (Indicates whether there was continuity in participants’ membership in a group throughout the program or if participants could switch between different groups) | NA |
| 18. Group size  (Reports the number of participants per group (average and/or range)) | 6, 9 |
| 19. Number of facilitators  (Reports the number of facilitators delivering the sessions, i.e., how many facilitators delivered each of the sessions) | 7 |
| 20. Continuity of facilitators’ group assignment  (Indicates whether there was continuity in facilitator’s assignment to a group throughout the intervention, i.e., if the same or different facilitator(s) delivered the sessions to each group of participants.) | NA |
| 21. Facilitators’ professional background  (Reports facilitators’ professional background, status as a non-professional, or relevant qualifications) | 7 |
| 22. Facilitators’ personal characteristics  (Reports relevant personal characteristics of the facilitators, i.e., who they were in terms of age, gender, ethnic or cultural background, education level, socio-economic status etc) | 7 |
| 23.Facilitators’ training in intervention delivery  (Reports what training in delivering the intervention the facilitators were provided with) | 7-8 |
| 24. Facilitators training in group facilitation  (Reports what training in group facilitation methods the facilitators were provided with, i.e., how to work with and facilitate groups.) | NA |
| 25. Facilitators’ materials  (Reports whether the facilitators were provided with materials and/or written instructions to be used to guide delivery of the sessions.) | 6-8 |
| 26. Facilitation style (Describes the intended style of, or approach for, the session delivery  and group facilitation) | 6-7 |
